# The Importance of Stroma and Stromal Sma Expression in Pancreatic Ductal Adenocarcinoma*

**DOI:** 10.5146/tjpath.2024.13521

**Published:** 2024-09-02

**Authors:** Gamze Akbas, Pelin Bagcı

**Affiliations:** Department of Pathology, Marmara University, School of Medicine, Istanbul, Turkey

**Keywords:** Pancreas, Cancer, Stellate cell, Tumor stroma, α-SMA, Angioinvasion

## Abstract

***Objective: *** Pancreatic stellate cells (PSC) have been defined to be the key players in pancreatic fibrogenesis and carcinogenesis. They undergo myofibroblast-like differentiation, express α-smooth muscle actin (α-SMA), and play a crucial role in injury and inflammation sites. This study aims to evaluate the relationship between α-SMA expression and histopathological parameters of pancreatic ductal adenocarcinoma (PDAC), and investigate their association with prognosis.

***Material and Methods:*** Eighty-one consecutive pancreatectomies diagnosed as usual pancreatic ductal adenocarcinoma were included. The stromal density was scored as loose, moderate, or dense, and α-SMA expression was evaluated immunohistochemically.

***Results and Conclusion: *** Mean survival was 19.6 months. Male gender, larger tumor diameter (>3.7 cm), and older age (>64 years) were identified as independent poor prognostic factors. Perineural invasion significantly effected survival. A statistically significant correlation was found between high α-SMA expression and the presence of angioinvasion (p=0.01). Stromal α-SMA expression in PDAC may help determine the risk of angioinvasion.

## INTRODUCTION

Pancreatic ductal adenocarcinoma (PDAC) ranks as the fourth leading cause of cancer-related deaths in males and the third in females (1,2). The primary reasons for its poor survival rates include low operability at the time of diagnosis and the resistance to conventional chemotherapy protocols. Hence, there is an urgent need for new targeted therapeutic approaches (1).

The stroma of cancers is a recent hot topic of research. Colorectal tumors are the first to be proven to have significant prognostic outcomes related to their stroma (3). Ueno et al. classified the desmoplastic reaction as mature, intermediate, or immature based on the presence of hyalinized keloid-like collagen and myxoid stroma at the extramural desmoplastic front. The prognostic power of this categorization in stratifying relapse-free survival was greater than any other conventional prognostic factors, including TNM stage, venous invasion, and tumor grade (4,5). Subsequently, Wang et al. applied an undefined scoring system using H&E and Masson’s trichrome stains for PDACs (6).

The stroma of PDAC constitutes more than 50% of the tumor mass and includes various components such as extracellular matrix (ECM), pancreatic stellate cells (PSC), fibroblasts, macrophages, blood and lymphatic vessels, pericytes, stem cells, and inflammatory cells. Recent research has focused on developing treatment strategies targeting stromal elements, particularly PSCs, which play a central role in pancreatic fibrogenesis (7–12). PSCs are star-shaped, vitamin A-storing cells, which comprise approximately 4% of all pancreatic cells and show a periacinar distribution in a healthy pancreas. In response to several injuries (such as cancer or inflammation), PSCs undergo an activation process via exhibiting a myofibroblastic-like phenotype, expressing α-smooth muscle actin (α-SMA). Later they become the key cells in pancreatic fibrogenesis (13,14).

In many studies, PSCs have been shown to be present since the early stages of preneoplastic transformation. PSCs are activated and exhibit myofibroblastic morphology during epithelial carcinogenesis. This observation can be explained by three hypotheses: 1) PSCs may interact with tumor cells from the early stages of carcinogenesis, 2) they may attempt to circumvent and confine tumor cells from early preneoplastic stages, or 3) PSCs may activate around genetically defective cells in an initially limiting manner, then synthesize elements of the extracellular matrix (ECM) to make the stroma more fibrotic. This fibrosis can lead to hypoxic conditions in subsequent stages, resulting in the release of reactive oxygen species and genetic instability in epithelial cells (15).

Although there are clinical (oncology, surgery) and preclinical (biochemistry, pharmacology) studies related to PDAC stroma in the literature (15–18), publications based on histopathological examinations are few and serve only to complement the gaps between these studies (6,19–21). This study aims to evaluate the relationship between α-SMA expression and the histopathological parameters of pancreatic ductal adenocarcinoma (PDAC), as well as to investigate the effects of these parameters on the prognosis.

## MATERIALS and METHODS

**Figure 1:**
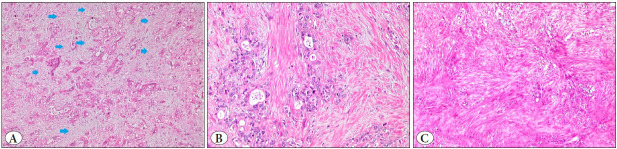
**A)** Myxoid or loose stroma of pancreatic ductal carcinoma. The periglandular area is pale, has less myofibroblasts, and looks edematous (Hematoxylin & Eosin, x10). **B)** Moderate or keloid-like stroma of pancreatic ductal carcinoma. Interrupted bands of dense eosinophilic keloid-like collagen are seen in the picture. This collagen is the product of activated myofibroblasts (Hematoxylin & Eosin, x40). **C)** Strong or mature stroma of pancreatic ductal carcinoma. Collagen replaced everything between and around the tumor cells. There are a few tumor cells at the top center of the figure (Hematoxylin & Eosin, x40).

All pancreatic resections examined in the Department of Pathology between 2011 and 2016 were re-evaluated retrospectively. All consecutive cases with a diagnosis of usual (classical or conventional) PDAC were included in the study (n=81). PDACs with nonconventional subtypes and those associated with intraductal papillary mucinous neoplasm (IPMN) or mucinous cystic neoplasm (MCN) were excluded. A Whipple resection had been performed in 62 cases, a total pancreatectomy in 6 cases, a distal pancreatectomy in 10 cases, and partial excisions in 3 cases. Information on histological tumor subtypes, grade, location, diameter, and lymphatic, vascular, and perineural invasion, as well as surgical margin status, was retrieved from pathology reports. After revision of all tumor slides, the pattern of stromal density was classified based on all hematoxylin and eosin (H&E) stained tumor slides as loose (myxoid), moderate (keloid-like), or dense (mature) according to the relevant literature (4–6). The definition of the final stromal score in these studies was not clear enough. Therefore, the scoring of the stromal pattern was based on the dominant density (≥ 50% of the total tumor stroma) for each case. Loose stroma was defined as a loose fibroblastic and myxoid stroma containing short, faint collagen fibers (Figure 1). Moderate density stroma was composed of haphazard bands of keloid-like collagen (Figure 1). Dense stroma was characterized by the dominance of mature, thick collagen fibers that resembled scar tissue (Figure 1). For statistical purposes, the stroma was grouped as mixed (loose + moderate) and dense. The best representative formalin-fixed paraffin-embedded (FFPE) tumor block was selected for immunohistochemistry. This block showed the dominant type of stromal density and did not contain necrosis.

### Immunohistochemical Analysis

The tumor blocks were sectioned at 4 µm thickness as positively charged slides. For the processes, the Leica Bond-Max automatic immunohistochemistry device was used. To block the endogenous peroxidase activity, the sections were incubated for 10 min with three steps of at room temperature. Antigen retrieval was achieved with tris-EDTA buffer (pH 8) for α-SMA. The slides incubated with α-SMA (1:200, Clone 1A4, Cell Marque) for 45 minutes at room temperature. After primary antibody, secondary antibody and poly-HRP solutions (Bond Polymer Detection Kit; DS9800, Leica) were applied in the device. Diaminobenzidine (DAB) was used as the chromogen for colored visualization of the antigens. The sections were then counterstained with hematoxylin and dehydrated with ethanol.

Two observers blinded to the clinical outcome interpreted the slides. α-SMA was expressed in the stroma only. Fine granular cytoplasmic staining was regarded as positive and the percentage (extensity) was scored as follows: 0 = 0-10%; 1 = 11-50%; 2 = 51-75%; 3 = 76-100%. The intensity of staining was graded as: 0 = none; 1 = weak; 2 = moderate; 3 = strong. An overall score for α-SMA was calculated as (extensity + intensity): negative = 0–1; low = 2-3; moderate = 4-5; high = 6 (Table 1).

**Table 1:** Scoring method of immunohistochemical staining for stromal α-SMA*

**Stromal score**	**Extensity (%)**	**Intensity**
0	0-10	None
1	11-50	Weak
2	51-75	Moderate
3	76-100	Strong
**Overall Stromal score** = Extensity score + Intensity score (negative = 0–1; low = 2-3; moderate = 4-5; high = 6)		

* α-SMA: alpha smooth muscle actin

### Statistical Analysis

Histopathologic and clinical variables were compared in terms of the SMA score. Categorical variables were compared using the Pearson Chi-Square test. Continuous variables were compared using the Kruskal-Wallis test. Cox-regression and Kaplan-Meier estimates were used to calculate the factors effecting survival. A multivariate Cox proportional hazards model was employed to identify independent prognostic factors. Statistical significance was set at a p-value of <0.05. All analyses were performed using jamovi (version 2.3) (22).

## RESULTS

### The Relationship Between Immunohistochemical and Clinicopathological Findings and Overall Survival (OS)

The mean age of the patients was 64, with a female/male ratio of 30/51. Most of the tumors were grade 2 (n=55, 68%) and were located in the head of the pancreas (n=67, 83%). The mean tumor diameter was 3.7 cm (range: 0.5- 10.2 cm). The distribution of pathological T stages according to the recent classification by the American Joint Committee on Cancer (AJCC) 2017 was T1/T2/T3/T4: 7/41/30/3, respectively. The N stages were N0/N1/N2: 20/23/37. Lymphatic invasion was found in 73 cases (90%). Vascular invasion was present in 55 cases (68%). Perineural invasion was found in 78 cases (96.3%). Forty-two cases (52%) had positive margins (R1) (Table 2).

**Table 2:** The baseline characteristics of the patients and histopathological findings (n = 81)

		**Mean (range) or n (%)**
Age		64 (34-85)
Gender	Female	30 (37)
	Male	51 (63)
Grade	Grade 1	8 (10)
	Grade 2	55 (68)
	Grade 3	18 (22)
Location	Head	67 (83)
	Corpus&tail	14 (17)
Tumor diameter (cm)		3.7 (0.5-10.2)
T (AJCC* 2017)	T1	7 (9)
	T2	41 (51)
	T3	30 (37)
	T4	3 (4)
N (AJCC 2017)	N0	20 (25)
	N1	23 (28)
	N2	37 (46)
Lymphatic invasion (present)		73 (90)
Angioinvasion (present)		55 (68)
Perineural invasion (present)		78 (96)
Surgical margin	R0	39 (48)
	R1	42 (52)

*AJCC: American Joint Committee on Cancer (T: Tumor stage, N: Lymph node stage)

**Figure 2:**
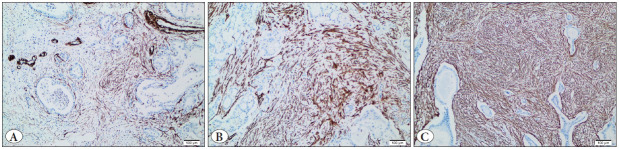
**A)** Low expression of α-SMA in the stroma of pancreatic ductal carcinoma (α-SMA antibody, x10). **B)** Moderate expression of α-SMA in the stroma of pancreatic ductal carcinoma (α-SMA antibody, x10). **C)** High expression of α-SMA in the stroma of pancreatic ductal carcinoma (α-SMA antibody, x10).

The total score of α-SMA immunohistochemistry was “low” in 2 patients (2%), “moderate” in 25 patients (31%), and “high” in 54 patients (67%) (Figure 2) (Table 3).

**Table 3:** Distribution of immunohistochemical staining for stromal α-SMA in the cohort

**Extensity Scores (n, %)**	1	1 (1)
	2	20 (25)
	3	60 (74)
**Intensity Scores (n, %)**	1	1 (1)
	2	13 (16)
	3	67 (83)
**Total Score (n, %)**	Low	2 (2)
	Moderate	25 (31)
	High	54 (67)

The stromal pattern exhibited mixed stromal density in 38 cases (47%), while 43 cases (53%) had a pure mature (dense) type stroma. Among the cases with pure mature (dense) stroma, 12 had low or moderate α-SMA scores, whereas 31 had high α-SMA scores. Of the cases with mixed stromal density, 15 had low or moderate α-SMA scores, and 23 had high α-SMA scores (Table 4).

**Table 4:** Distribution of stromal pattern and α-SMA scores

	**Stromal Pattern**	
**α-SMA Scores**	Mixed stroma (n=38, 47%)	Pure mature (dense) stroma (n=43, 53%)
Low + Moderate α-SMA score	15	12
High α-SMA score	23	31

**Figure 3:**
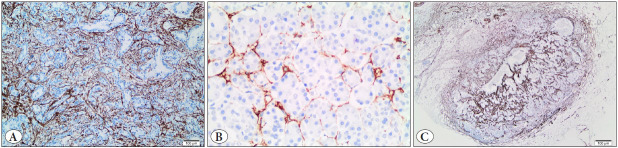
**A)** α-SMA staining was stronger at the immediate borders of the glandular tumoral component, and the interglandular area was less dense (α-SMA antibody, x10). **B)** Activated pancreatic stellate cells stained with α-SMA highlighting the normal perilobular area (α-SMA antibody, x40). **C)** Lymph node metastasis of a pancreatic ductal carcinoma. The same strong staining pattern was also seen in metastatic areas (α-SMA antibody, x10).

α-SMA was found to be strongly positive around the glandular tumoral component in 56 cases (Figure 3), as well as around the normal perilobular area in 24 cases (Figure 3). Staining was also stronger in peritumoral chronic pancreatitis in 27 cases. Additionally, the stromal components of metastatic foci in lymph nodes were positive for α-SMA in 12 cases (Figure 3).

The total scores of α-SMA did not reveal any significant correlation when compared with other clinicopathological parameters. However, α-SMA levels were found to be higher in patients with angioinvasion than in those without angioinvasion (p = 0.01) (Table 5).

**Table 5:** Relation of α-SMA and other pathological parameters

**Variables**	**n**	**High** **α-SMA Score** **n=54**	**Low+Moderate α-SMA Score** **n=27**	**p-values**
Gender: Male	81	31	20	p=0.14^1^
Age (mean)	81	62.5	68.0	p=0.20^2^
Grade	81			p=0.87^1^
1		6	2	
2		36	19	
3		12	6	
Location	81			p=0.06^1^
Body		2	5	
Distal		4	3	
Head		48	19	
Tumor Diameter (cm, mean)	81	3.5	3.5	p=0.83^2^
pT	81			p=0.45^1^
T1		4	3	
T2		28	13	
T3		21	9	
T4		1	2	
pN	80			p=0.25^1^
N0		12	8	
N1		13	0	
N2		28	9	
Lymphatic invasion (Present)	81	49	24	p=0.79^1^
Angioinvasion (Present)	81	42	13	**p=0.01^1^**
Perineural invasion (Present)	81	53	25	p=0.21^1^
Positive surgical margin (R1)	81	28	14	p=1.00^1^
Higher grade (Grade 3)	81	12	6	p=1.00^1^
Higher T stage (T3+T4)	81	22	11	p=1.00^1^

^1^Pearson. ^2^Wilcoxon. (p<0,05)

The mean overall survival (OS) was 19.6 months (range: 2-107 months), with a median survival time of 12 months. Male gender was associated with worse OS [HR: 1.51, 95% CI: 0.94-2.41, p=0.089]. Patients older than 64 years of age were associated with a worse prognosis [HR:1.02, 95% CI: 1.00-1.05, p=0.059)]. Patients with positive surgical margins had significantly worse OS [HR: 1.76, 95% CI: 1.11-2.78, p=0.016]. The presence of perineural invasion had a statistically significant negative effect on OS [HR: 4.30, 95% CI: 1.04-17.78, p=0.044]. The presence of angioinvasion was associated with worse outcomes [HR: 1.45, 95% CI: 0.89-2.35, p=0.136], and lymphatic invasion also worsened OS [HR: 1.23, 95% CI: 0.59-2.58, p=0.576]. Tumors located in the head of the pancreas showed worse survival compared to those in the distal and body locations [HR: 0.91, 95% CI: 0.41-1.99, p=0.809]. Tumors larger than the mean tumor diameter (3.7 cm) showed worse prognosis [HR: 1.33, 95% CI: 1.11-1.60, p=0.002], although this did not affect higher tumor stages. Tumors with higher stages (T3 and T4) had a worse prognosis [HR: 1.42, 95% CI: 0.90-2.25, p=0.132]. N2 cases had a worse prognosis compared to N1 cases [HR: 1.39, 95% CI: 0.79-2.45, p=0.259]. Tumors with low+moderate SMA scores had a worse prognosis compared to high SMA scores [HR: 1.20, 95% CI: 0.75-1.92, p=0.457)] (Table 6).

**Table 6:** Multivariate survival analysis

**Dependent: Survival**	**Levels**	**All**	**HR (univariate)**	**HR (multivariate)**
Age	Mean (SD)	63.8 (9.8)	1.02 (1.00-1.05, p=0.059)	1.03 (1.00-1.05, p=0.038)
Gender	Female	30		
	Male	50	1.51 (0.94-2.41, p=0.089)	1.88 (1.07-3.31, p=0.029)
Surgical Margin	R0	39		
	R1	41	1.76 (1.11-2.78, p=0.016)	1.63 (0.97-2.76, p=0.066)
Perineural Invasion	Absent	3		-
	Present	77	4.30 (1.04-17.78, p=0.044)	3.76 (0.65-21.69, p=0.139)
Angioinvasion	Absent	26		
	Present	54	1.45 (0.89-2.35, p=0.136)	1.08 (0.57-2.06, p=0.806)
Lymphatic Invasion	Absent	8		
	Present	72	1.23 (0.59-2.58, p=0.576)	0.58 (0.20-1.70, p=0.320)
Location	Body	7		
	Distal	7	0.97 (0.34-2.80, p=0.962)	0.71 (0.23-2.25, p=0.567)
	Head	66	0.91 (0.41-1.99, p=0.809)	1.62 (0.67-3.91, p=0.282)
Tumor Diameter	Mean (SD)	3.7 (1.4)	1.33 (1.11-1.60, p=0.002)	1.75 (1.22-2.53, p=0.003)
T Stage	T1+T2	47		
	T3+T4	33	1.42 (0.90-2.25, p=0.132)	0.49 (0.18-1.35, p=0.168)
N Stage	N0	20		
	N1	23	1.24 (0.67-2.29, p=0.492)	1.40 (0.69-2.84, p=0.355)
	N2	37	1.39 (0.79-2.45, p=0.259)	1.15 (0.57-2.29, p=0.697)
Sma Score	High	53		-
	Low + Moderate	27	1.20 (0.75-1.92, p=0.457)	1.28 (0.71-2.30, p=0.414)

Older age (>64 years), male gender, and larger tumor diameter (>3.7 cm) were found to be independent factors in multivariate analysis (with p-values of 0.038, 0.029, and 0.003, respectively). However, tumor diameter did not have a significant effect on the T stage of the tumor. Surgical margin status approached significance but was not an independent factor (p=0.066) (Table 6).

### Oncological Follow-Up

Forty-five cases were followed up at our institution. Two of them received neoadjuvant therapy: one received FOLFIRINOX, and the other received gemcitabine in combination with radiotherapy. Adjuvant therapy was administered to 45 patients, with two receiving FOLFIRINOX, 41 receiving gemcitabine, and two receiving a combination of capecitabine and gemcitabine.

Two cases that received neoadjuvant therapy had high α-SMA scores, with lifetimes of 37 and 42 months, respectively. Among the cases treated with adjuvant FOLFIRINOX, two had high α-SMA scores, with lifetimes of 16 and 37 months. Forty-one cases treated with adjuvant gemcitabine had moderate or high stromal α-SMA scores, with a mean lifetime of 24.7 months (range: 3-107 months) (Table 7). Two cases treated with the adjuvant gemcitabine and capecitabine combination had moderate-high SMA scores and the lifetimes were 32 and 10 months respectively. Due to the small number of cases treated with FOLFIRINOX (only two), and combination therapies (only two), the α-SMA scores could not be compared for different therapy regimens.

**Table 7:** Therapy types and SMA score distribution

**n=45**	**Chemotherapy Type**	**SMA Score**	**Lifetime (months)**
Neoadjuvant CT n=2	1- Folfirinox 1- Gemcitabine	High High	37 42
Adjuvant CT n=45	2- Folfirinox	High	16 and 37
	41, Gemcitabine 2, Capecitabine + Gemcitabine	Moderate/High	Mean=24,7(3-107) 32 and 10

Sixteen cases experienced distant metastasis, with 11 of them spreading to the liver or lungs. Additionally, 24 cases had local recurrence. All of these cases with metastasis or local recurrence had moderate or high α-SMA scores in their primary tumor.

## DISCUSSION

The stroma of colorectal tumors has been the first to be proven to significantly influence the prognostic outcome. Ueno et al. classified the desmoplastic reaction in colon tumors as mature, intermediate, or immature based on the presence of hyalinized keloid-like collagen and myxoid stroma at the extramural desmoplastic front. The prognostic power of this reaction categorization in stratifying survival was superior to that of any other conventional prognostic factors such as TNM stage, venous invasion, and tumor grade (4,5).

Bever et al. utilized computer-based image analysis of collagen-stained slides to calculate a specific stromal density score for 66 PDAC cases who underwent adjuvant chemotherapy. However, they did not observe any correlation with prognosis (23). Although it appeared to be an accurate method for calculation, this was reported as an expensive and non-reproducible method for scoring the stroma of PDAC.

Afterward, Wang et al. applied a modified method using hematoxylin-eosin with Masson’s trichrome, and α-SMA for PDACs (6). They found that high stromal density was associated with a significantly better clinical outcome compared to patients with intermediate or low stromal density in multivariate analysis. The stromal pattern was defined as an independent poor prognostic factor (6).

Methods for examining stromal density vary widely in the literature, ranging from eyeballing to computer-based image analysis. We believe that a specific, reproducible, easy, and cost-effective method for assessing stromal density needs to be established through larger studies.

Pancreatic stellate cells (PSCs) become activated in situations such as inflammation and carcinogenesis, leading them to express α-SMA (13,14,24). We also observed stronger α-SMA staining in peritumoral chronic pancreatitis, which supports the thesis that pancreatic stellate cells (PSCs) are triggered by chronic inflammation (not shown here).

Fujita et al. studied 109 resection specimens of PDACs and discovered that high levels of α-SMA mRNA were associated with a worse prognosis. However, it is worth noting that the adjuvant chemotherapy regimens in this study were heterogeneous (19). Similar results were obtained in the CONKO-001 study (25). However, a “tissue microarray” assay was used for immunohistochemical staining in this study, which we believe may not adequately demonstrate the heterogeneous distribution of stromal α-SMA. The therapy regimens were classified as treated with or without gemcitabine, and the antibody clone used was different from ours (Clone: M0874). In contrast to these studies, Özdemir et al. utilized 53 genetically altered mice with decreased α-SMA-expressing myofibroblasts. The response to injury was a decrease in the amount of extracellular matrix (ECM), leading to increased tumor progression. The final result was a worse tumor prognosis (26). Erkan et al. proposed an “activated stromal index” combining high levels of α-SMA and low levels of collagen, and found a relationship with a worse prognosis. However, they could not reveal a significant effect of α-SMA levels alone on survival (20). Wang et al. found that the effect of α-SMA expression on the survival of 145 resected PDACs treated with adjuvant gemcitabine was significant in univariate analysis, but they did not observe any significant results in multivariate analysis (6).

In our study, there was no significant association between the immunohistochemical expression of α-SMA and overall survival (univariable/multivariable: p=0.457/p=0.414). Only 45 cases were followed up in our institution, with 43 receiving gemcitabine-based adjuvant chemotherapy and 2 receiving FOLFIRINOX. Among them, 24 experienced recurrence, and the drug types chosen for recurrence varied (gemcitabine, XELOX, FOLFOX, paclitaxel, etc.). Since most of the cases were treated with the same first-line gemcitabine-based regimen, statistical analyses to assess the relationship between the dominant stromal pattern and/or α-SMA density with response to therapy could not be performed.

Lymph nodes were not within the scope of our study; however, in some cases (n=12), they were present on the slides selected for immunohistochemistry. We observed that the stromal components of metastatic foci in lymph nodes were also strongly positive for α-SMA, indicating that the tumor carries these stromal features wherever it spreads. We believe that examining the stroma in metastatic areas such as the liver, lung, and lymph nodes should be the next step.

The major outcome of our study was the significant correlation between higher α-SMA scores and the presence of angioinvasion (p=0.01). Previous similar studies did not report this kind of correlation (6,19). Pancreatic stellate cells are also recognized to have angiogenic or antiangiogenic effects in both the early and late stages of the disease (27). The presence of such an association may suggest that tumors with higher α-SMA scores are at a higher risk for vascular invasion and local recurrence and/or distant metastasis. This result could be valuable in predicting the metastatic capacity of the tumor. However, due to the small size of our cohort, this preliminary finding did not reach statistical significance in the multivariate analysis (p=0.136).

We believe that the disparate results regarding the effects of α-SMA on survival in the literature are primarily attributable to the retrospective nature of the studies, as well as variations in tissue types, tissue sizes, antibody clones, and the quality of the cohorts. To mitigate these discrepancies, prospective studies and clinical trials should be designed based on analyses and scoring of stromal α-SMA. Additionally, our study primarily focuses on α-SMA expression at the protein level. Integrating molecular analyses, such as gene expression profiling or mutation status, could provide deeper insights into the underlying biological mechanisms driving the observed associations.

## Conflict of Interest

The authors declare that they have no conflict of interest for this article.

## Funding

The financial support was received from the Marmara University Committee of Scientific Research Project (BAPKO) (Project No: SAG-C-TUP-110915-04).

## Ethics Approval

Ethical approval was obtained from the local human ethics committee at Marmara University Institute of Health Sciences (Protocol number: 88-15).
